# Digital inclusion, online participation and health promotion: promising practices from community-led participatory journalism

**DOI:** 10.1177/17579759221126150

**Published:** 2022-10-26

**Authors:** Kate Mulligan

**Affiliations:** University of Toronto, Canada

**Keywords:** health promotion, community action, communication (including social marketing, education campaign, media communications), advocacy (including media advocacy), social networks, determinants of health, e-health, equity/social justice

## Abstract

A growing body of evidence demonstrates that digital inclusion mediates access to other social determinants of health – directly, through access to and literacy in technologies or services, and indirectly, by supporting people’s capacity to participate fully and equitably in civic and cultural life online. Novel approaches to community-led participatory journalism, in which people from equity-deserving communities are supported to tell their own stories, suggest promising practices for online civic engagement as an emerging approach to promoting health though digital inclusion.

Around the world, people are spending increasing amounts of time online – so much that we are beginning to see arguments for the recognition of digital ‘superdeterminants’ as mediators of access to other social determinants of health (1). In response, health promotion has begun to focus on meeting five goals for *digital inclusion*: ensuring the equitable distribution of 1) reliable Internet access, 2) appropriate devices, 3) digital literacy, 4) technical support, and 5) opportunities for online participation, so that ‘all individuals and communities, including the most disadvantaged, have access to and use of Information and Communication Technologies’ (2). To date, initiatives for digital inclusion and health equity have primarily focused on *access* to technology and supports, in part because digital technologies and connections are inequitably distributed – disproportionately excluding people living in rural communities, Indigenous people, older adults and people living in the Global South (3). Some initiatives have focused on digital health *literacy*, supporting people’s understanding and use of digital tools to gain ‘greater autonomy and control over health decision making’ (4) and improve access to other determinants of health – including food, housing, education, jobs and government services, which may be available only through online sources. Digital health literacy interventions in clinical and community settings – such as targeted multimedia training programmes in doctors’ offices, libraries and early learning programmes – show promise for the improvement of health and inclusion across social gradients (5–7).

Fewer health promotion initiatives, however, have yet to focus on supporting the fifth component of digital inclusion: online participation and collaboration. As public and civic discourse increasingly moves to online social networks, there is a risk that people and communities facing health inequities will face further exclusion from vital online conversations shaping public policy and public health practice. In response, health promoters and researchers have begun to call for ‘online content designed to enable and encourage self-sufficiency, participation, and collaboration’ in these spaces (5). To date there are few examples of targeted supports for online participation from within public health practice; however, there are strong examples from other fields that hold promise for health promotion. The purpose of this commentary is to share promising examples of participatory online health promotion from community-led participatory journalism, in which people from equity-deserving communities are supported to tell their own health-related stories in online publications and on online social networks. These examples demonstrate the importance of three key components of inclusive online engagement that are relevant for inclusive digital health promotion: financial and training supports for local participation and engagement, strengths-based approaches to understanding and portraying communities, and mechanisms for connecting the stories of seldom-heard communities with decision makers.

Under mainstream approaches to media, communities facing health inequities are often ignored (8), see community deficits emphasized over strengths (6) or have their knowledge extracted without reciprocity (9). As part of a broader political economy rooted in concentrated media ownership and weak regulation, corporate-owned mainstream media approaches tend to privilege feedback loops involving already-powerful people, politics and perspectives (10). Mainstream media remains important for health promoters and public health practitioners who need to reach decision makers and the public through earned reporting, opinion papers and guest speaking. It also provides an important democratic safeguard for accountability and transparency respecting the explicit policies, practices and resource flows important for systems change (11). However, it cannot fully challenge the political dynamics that produce health inequity, polarization and unsustainability from within.

The primary alternative to date has been citizen journalism, in which ordinary people become the main creators and distributors of news (12). Citizen journalism, enabled by growing populational access to smartphones and social media technologies, emerged first as a form of activism in oppressive and under-resourced environments. Examples include Hong Kong’s democracy movement (13), the 2010 Arab Spring (14) and Indigenous journalism within colonial states (15). In 2021, teenager Darnella Fraser was awarded a Pulitzer Prize for ‘courageously recording the murder of George Floyd, a video that spurred protests against police brutality around the world, highlighting the crucial role of citizens in journalists’ quest for truth and justice’ (16). As a form of civic participation, citizen journalism has been linked to expressions of belonging and engagement in local neighbourhoods and communities (17). Despite its continued relevance, however, citizen journalism provides few to no resources or support for online engagement. Its impact relies on the existing resources of its creators and the timeliness of its content. A more sustainable civic journalism requires more systematic support for content creators and distributors.

Community-led participatory journalism provides such support. It is a form of supported civic journalism in which ‘community members can practice journalism themselves for the community in which they reside’ (18). It is distinct from mainstream, corporate local journalism and from traditional citizen journalism in that it does not expect that participants are already able to tap existing resources, knowledge, social networks or privileges in order to participate. Instead, community members are provided with the training, support and networks they need to participate in online discourses impacting their own lives by engaging in storytelling and reporting online – a redistribution of the foundations of online participation. By building and supporting capacity, and transferring resources toward new ways of engaging online, community-led participatory journalism shares power rather than concentrating it in existing networks, involves more of the community in dialogue, and builds long term capacity rather than extracting community knowledge for transactional reports and stories. For institutions struggling to create opportunities for collaboration and access (19), this is the work of digital inclusion: the direct and supported engagement, voices and stories of people facing health inequities, in online networks. Community-led participatory journalism also generates and shares new public knowledge rooted in the strengths of equity-deserving communities. Investment in community-led participatory journalism offers access to the local expertise necessary for decision makers and public health practitioners, among others, to solve complex problems such as community trust in vaccination or context-specific approaches to climate change (20).

Across diverse and context-specific models, community-led participatory journalism has three foundational components: support for local participation and engagement, a strengths-based approach to understanding and portraying communities, and a mechanism for connecting the stories of seldom-heard communities with decision makers. For example, the Toronto-based online magazine *The Local*, which began as an innovation project of the city’s largest hospital network, employs community-based journalists and provides training and mentorship to an annual cohort of journalism fellows from underrepresented and marginalized communities. It aims to help them report on urban health and social stories ‘from corners of the city that are too often ignored or misunderstood’, ‘from perspectives too often ignored’ (21). *The Local*’s coverage of COVID vaccination in underserved communities earned national awards and influenced government policy. Two weeks after *The Local* published a community-strengths story on vaccination, the Ontario government made the decision to allocate 50% of vaccines to hotspot postal codes: ‘“If you’re in doubt about whether vaccines should be prioritized to ‘hot spots’, read this account,” said a former Minister of Health’ (22). The story’s sub-heading reads: ‘When the pandemic hit Peel, it wasn’t the government that stepped in, but an army of citizens that mobilized to feed their neighbours, set up pop-up clinics, and demand better for their community’ (23).

Chicago-based non-profit City Bureau provides similar training through its Civic Reporting Programs (24). It also trains, supports and pays local ‘documenters’ to monitor local government by reporting on hundreds of meetings that would otherwise go unrecorded, creating a detailed digital repository for civic engagement and accountability. To date the programme has trained over 100 BIPOC (Black, Indigenous, People of Color) reporters and over 800 paid public documenters, producing more than 300 stories that resulted in ‘better informed communities and local policy change.’ The programme measures impact according to metrics for equity, digital participation, community building and collective change (25). City Bureau stories also focus on community strengths; the subheading of one story reads: ‘Tired of city officials’ promises to bring more grocery retailers to their neighbourhoods, urban farmers and local organizers aren’t waiting around’ (26).

The Lancaster-based COVID Other Front Line Alliance provides support in the form of co-journalists, who work with ‘street journalists and bloggers’ from around the world to help them share their stories online while ensuring the community journalist maintains copyright under a creative commons licence (27). The Alliance mobilizes action based on the testimonies of people impacted by structural inequities and the stories of people taking local action to protect community health. This work has been described as a key component of a ‘social vaccine’ of government and civil society action that can create the conditions for global health and equity (28). The story cited here reads: ‘The deeper we got into “lockdown” here in the UK the more I noticed the hidden abundance in my neighbourhood and with my neighbours. I wrote this at the end of week 5 of lockdown, and I believe the Covid-19 pandemic is shining a light upon the abundance in our community, thinking of others and sharing what they have’ (29).

The examples explored here demonstrate the strong potential of community-led participatory journalism as a tool for health promotion in an era of digital superdeterminants of health: a way to bridge digital divides that goes beyond digital access and digital literacy to support and sustain online participation as a key facet of digital inclusion. Three concrete actions offer particular promise to health promoters seeking to support community participation in the digital public sphere: provide financial and training supports for online leadership and participation, start with strengths-based orientations to community health, and connect communities and decision makers on what matters to communities.

Reviews from media and communications studies support the promise of these practices, suggesting that community journalism can indeed support sense of belonging and community building (30), and that meaningful community participation is better supported by civic organizations than commercial ones (31,32). However, these approaches have yet to be adapted and evaluated in the context of health promotion; a recent review of health promotion initiatives for public participation in health decision making found that most such initiatives lack transparency and are under-theorized (33). The gap between these promising practices and the evidence base demonstrates a clear need for additional practice-based and theoretical research to better understand the impacts and mechanisms of action of community-led participatory journalism on health promotion and the determinants of health.
